# Antioxidant Role of High-Density Lipoprotein

**DOI:** 10.3390/antiox15020186

**Published:** 2026-02-02

**Authors:** Paul N. Durrington

**Affiliations:** Cardiovascular Research Group, Faculty of Biology, Medicine and Health, University of Manchester, Manchester M13 9NT, UK; pdurrington@manchester.ac.uk; Tel.: +44-161-275-1201; Fax: +44-161-275-1183

## 1. Introduction

Low-density lipoprotein (LDL) is the primary cholesterol-carrying lipoprotein in the circulation, where it is a significant contributor to atherosclerosis and delivers cholesterol to the tissues [1]. The role of high-density lipoprotein (HDL) is, however, far from certain.

## 2. HDL in Lipid Transport

In the human foetus, an apolipoprotein E-rich HDL (apoE-rich HDL) delivers cholesterol to cells via the LDL receptor (also known as the apoB100/apoE receptor, because it binds both the apolipoprotein B100 (apoB100) and apoE ligands) [1,2]. HDL is the dominant lipoprotein in the foetus and in cord blood, but within a few days of birth, both the apoE content of HDL and its contribution to serum cholesterol diminish as cholesteryl ester transfer protein (CETP) activity and circulating LDL cholesterol concentrations rise. LDL is then often regarded as being the dominant lipoprotein species in the blood circulation, supplying cholesterol, particularly to growing and repairing tissues and to those with a high requirement for steroidogenesis [1]. In the human CNS, however, apoE-rich HDL continues as the major cholesterol-carrying lipoprotein in the CSF [3]. Nonetheless, as clinicians are well aware, the serum HDL cholesterol concentration in humans, except in neonates, is almost invariably less than that of LDL. However, in terms of the serum concentration of their principal proteins, in healthy adults, the apolipoprotein A1 (apoA1) of HDL typically exceeds that of apoB100 in LDL. Furthermore, because of its smaller size, HDL crosses capillary endothelia more readily than LDL and thus, in terms of concentration of protein and of cholesterol, is the dominant lipoprotein in the tissue fluid. HDL cannot, therefore, be regarded as some minor lipoprotein with little significance. Alcover and colleagues summarise much of the current thinking about the formation and metabolism of HDL in Contribution 1 of this volume.

In many animals, apoE-rich HDL rather than LDL continues to be the major supplier of cholesterol throughout life [4]. In the rare human disorder abetalipoproteinaemia, where apoB is not produced and LDL is absent, apoE-rich HDL continues to supply cholesterol to tissues, albeit not compensating entirely for the absence of LDL, allowing red blood cells to become acanthocytes (due to lack of cholesterol in their cell membranes) and not fulfilling the role of apoB48 in the intestinal absorption of dietary fat and fat-soluble vitamins [1]. Clearly, in most human circumstances, however, HDL is no longer apoE-rich and after birth has no, or very little, role in delivering cholesterol to the tissues. This is strongly supported by the fact that most of the LDL receptors outside the liver and adrenals are likely to be saturated once the concentrations of LDL cholesterol levels exceed 2 mmol/L [5], which is the case in most adult humans, even when receiving a traditional Asian diet. The typical higher adult levels of LDL cholesterol appear to serve no useful purpose but do predispose to ASCVD and impose a requirement for the cholesterol transported out of the liver to be returned. HDL was for many years considered to be crucial in this futile, and potentially harmful, vicious cycle. This was thought to explain the inverse relationship between HDL cholesterol and ASCVD incidence reported in epidemiological studies [6]. However, as we discuss in Contribution 2, although the apparently protective role of HDL against ASCVD is indisputable, evidence that this is due to HDL playing some rate-limiting function in reverse cholesterol transport has not really been forthcoming, and the contention is fraught with anomalies.

## 3. HDL as a Protective Molecule

The investigation of HDL proteomics has revealed more than 100 protein components [7]. Many of these are present in only trace amounts, but those which stand out both in concentration and as being involved directly in pathogenesis or in susceptibility to a variety of diseases are apoA1, apolipoprotein A11 (apoA11), clusterin (also known as apolipoprotein J (apoJ), serum amyloid A (SAA), and lecithin (cholesterol acyltransferase (LCAT), myeloperoxidase and paraoxonase 1 (PON1)). Phospholipase A2 is present on HDL, but is mainly located on LDL. Its apparent activity on HDL is largely due to the presence of PON1 [8]. ApoA1 is an essential structural component of HDL and acts as a strong detergent, permitting the transport of lipids otherwise highly insoluble in water. It is a cofactor of LCAT, also located on HDL, which, particularly in humans, converts gram-range quantities of free cholesterol to cholesteryl esters, usually transferring to cholesterol the fatty acyl group in the Sn-2 position of phosphatidyl choline, in which HDL is rich [1]. The product of this reaction is the highly toxic lysophosphatidyl choline, which, if released into the circulation, would cause widespread damage to cell membranes. However, it remains safely confined to HDL, whence it is cleared by the liver. Although LCAT deficiency, whether genetic or acquired, for example, in extreme liver failure, is associated with disease complications associated with cholesteryl ester deficiency, its overexpression is not a cause of ASCVD [9]. However, its activity does serve to illustrate the capacity of HDL to protect the body from exposure to harmful substances. PON1 was discovered as a defence against synthetic organophosphate toxicity. This role also involves defending against exposure to naturally occurring organophosphates, such as those produced by blue-green algae. PON1 has proved to have an enormous range of hydrolytic substrates, many potentially harmful, such as lipid peroxides produced as a consequence of oxygen species reacting with phospholipids, an essential component of cell membranes and lipoproteins [10,11]. The evolution of aerobic respiration, although it brought many benefits in terms of the rate at which energy can be generated and exerted, was a hazardous business as reactive oxygen species leaking from processes, such as respiratory chain phosphorylation or from mechanisms to transport oxygen, are damaging to other biochemical processes, proteins, and tissues. Paraoxonases may well have evolved as lactonases before organisms developed aerobic metabolism. This lactonase activity may well continue to this day to contribute to their protective function against tissue damage by naturally generated lactones. Jakubowski and colleagues discuss this in detail in Contribution 3. However, this is not to deny their contribution to the antioxidative role of HDL in the case of PON1 and the intracellular antioxidative role of another family member, paraoxonase 2.

In Contribution 4, attention is drawn by Birkowitz-Fiebich and colleagues to the lower levels of lipid peroxides in HDL when higher HDL levels prevail, particularly in women.

The presence of clusterin (apoJ) on HDL, along with PON1, immediately suggests a major role for HDL in protecting the outer cell membranes and potentially their contents against damage that might otherwise result in disease or worsening of disease. Clusterin is a glycoprotein that protects against the toxicity of effete and damaged proteins [12]. We observed the increasing localisation of apoA1, PON1, and clusterin within atheromatous plaques as they progressed [13].

## 4. HDL in Disease Prevention

The notion that HDL has a protective role is further strengthened when one considers the wide range of diseases associated with low PON1 activity. In conditions predisposing to ASCVD and its partner in crime, aortic stenosis due to atheromatous narrowing of a congenitally bicuspid valve, discussed by Kwatiatkowska and colleagues in Contribution 5, HDL has a clearly emerging role. The same may be said in metabolic syndrome, hepatic steatosis, and diabetes [14]. Acute and chronic infections are also associated with decreased antioxidative capacity of HDL, as signalled by lower activity of PON1 [14,15], discussed by Reichert et al. in Contribution 6 in the case of HIV infection. The same may be true of several psychiatric and neurological conditions [14,16], such as depression, bipolar disorder, schizophrenia, and neuropsychiatric symptoms due to industrial organophosphate exposure [17], cerebral infarction, Alzheimer’s disease, Parkinson’s disease [14], and multiple sclerosis. The latter is reviewed in Contribution 7 by Damiza-Detmer and co-workers. Neoplastic disease is also associated with decreased HDL levels and low PON1 activity [14], as it now seems may also be the case for mast cell tumours of dogs reported by Ginoudis et al. in Contribution 8. Inflammatory arthritides and other acute and chronic inflammatory disorders are also associated with lower PON1 [18].

In many diseases associated with decreased serum PON1 activity, the circulating HDL particle size is decreased in comparison to HDL in health. There is decreased PON1 activity. This may be substrate-specific, but it also applies when a substrate like phenyl acetate, to which genetic isoforms of PON1 display equal activity, is used in the PON1 assay. Nonetheless, individuals may also display varying rates of hepatic synthesis and secretion, contributing to lower levels. However, a major cause of diminished PON1 activity appears to be the result of compositional change in HDL in diseases associated with low PON1 activity. Thus, in HDL, apparently predisposing to disease, myeloperoxidase is frequently elevated [10,19]. So also are apoA11 and SAA, both of which displace PON1 from HDL [10]. On the other hand, apoA1, essential for PON1 to be active against more hydrophobic substrates, is diminished. This gives rise to the concept of pro-inflammatory, pro-atherogenic HDL in disease states and anti-inflammatory, anti-atherogenic HDL in health. PON1 is an easily measured indicator of the type of HDL likely to be present (see later). The anti-inflammatory, anti-atherogenic type of HDL is also more effective in promoting the uptake of cholesterol from macrophage cells in tissue culture [20]. This cholesterol efflux capacity is related to ASCVD risk [21]. However, this is not to gainsay the earlier comments in this introduction about HDL and how little it is responsible for reverse cholesterol transport, because most of the cholesterol secreted by the liver in VLDL is cleared from the circulation with LDL via hepatic LDL receptors and thus short-circuits the tissues in which, in any case, LDL receptors are down-regulated. For cholesterol to cross the arterial endothelium (as opposed to capillary endothelia) and be incorporated into atheromatous lesions, LDL must first be modified, say by oxidation and/or glycation, so that it binds to scavenger receptors (see Contribution 2). Whilst this process is clearly related to the height of habitual circulating LDL cholesterol levels, not even in the most hypercholesterolaemic individuals can it be said to be a major fate for the enormous quantities of LDL cholesterol produced each day in humans [5].

## 5. Antioxidant Role of HDL

That high-density lipoprotein (HDL) could prevent the oxidative modification of proteins was discovered as the result of experiments involving the incubation of low-density lipoprotein with and without HDL under oxidising conditions. In the absence of HDL, changes in the physical properties of LDL, such as electrophoretic mobility, occurred, which were abolished or ameliorated [10]. Later, it was found that co-incubation of HDL with LDL in the presence of an oxidising agent such as copper ions prevented the accumulation of lipid peroxidation products [22] and the fragmentation of the apolipoprotein B component of LDL. This apoB fragmentation could otherwise lead to failure of LDL binding to the physiological LDL receptor of Goldstein and Brown, but rapid uptake of LDL by endothelial and macrophage cells via their scavenger receptors [11,23]. It was further realised that this cellular uptake could hasten the passage of LDL across the arterial endothelium (transcytosis) into the subintima, and once there, macrophage foam cell formation (see Contribution 2). Conjugated diene formation is the first stage in lipid peroxidation when unsaturated fatty acyl groups are exposed to oxygen-free radicals. The formation of alternating double/single carbon bonds is the consequence of hydrogen abstraction from unsaturated fatty acids present in phospholipids. It is easy to detect by ultraviolet spectroscopy and is opposed by fat-soluble vitamins present on LDL [24]. In contrast to fat-soluble antioxidant vitamins, which had largely failed to prevent atherosclerosis in clinical trials, the protection of LDL against oxidative modification by HDL and PON1 was unrelated to conjugated diene formation but was associated with decreased generation of lipid peroxidation products occurring downstream [10,11,24]. It is likely that this is due to hydrolytic removal of peroxidised unsaturated acyl groups at position Sn-2 on phosphatidyl choline and on cholesteryl esters by PON1 and sequestration by HDL of lysophosphatidyl choline and the ketone and aldehyde products of the breakdown of the peroxidised fatty acyl groups released. Some evidence suggests that the latter may be metabolised to harmless fatty acids [11,25], but in any case, they, together with lysophosphatidyl choline, will be removed during the passage of HDL through the liver.

## 6. The Place of HDL in the Clinic

The measurement of HDL cholesterol in clinical practice, usually to enter into an ASCVD risk algorithm as the term total serum cholesterol to HDL cholesterol concentration ratio or non-HDL to HDL cholesterol concentration ratio, is well established [26]. Monitoring responses to lipid-lowering medication by non-HDL cholesterol is also widely recommended, although there is an increasing tendency for guidelines to recommend direct apoB assays for this purpose [26]. There is a loose correlation between PON1 activity and HDL cholesterol, most evident in the general population [27]. However, there is discordance between the two, mostly in diabetes, established ASCVD, and dyslipidaemia [19,28,29]. The question thus arises whether anything of clinical value could accrue from measuring PON1 rather than, or in addition to, HDL cholesterol as a marker of whether the HDL present is pro-inflammatory and pro-atherogenic and therefore deficient in its antioxidant capacity. In general, this is controversial. However, in diabetes and established ASCVD, knowledge of PON1 activity may add to the assessment of the likelihood of future ASCVD events [28,29]. This might signal the need for more intensive intervention with, for example, lipid-lowering, antihypertensive, or anti-coagulant treatment. Given the range of diseases associated with decreased PON1 activity, its possible value in determining therapy in these conditions should also be explored.

Of course, there is also the issue of whether increasing serum PON1 activity could have therapeutic value. Few traditional therapeutic approaches to raising PON1 levels have emerged thus far. Extracts of pomegranate may, however, lead the way forward [30]. There has also been considerable interest in the production of PON1 by recombinant DNA technology, largely to combat or ameliorate organophosphate toxicity due to military and industrial exposure or to self-harm [17]. The biopharmaceutical versions of PON1 are modified in their manufacture to be water-soluble, which means they have therapeutic potential against water-soluble PON1 substrates, but not those for which the lipid solubility of naturally occurring PON1 embedded in HDL is critical for hydrolysis. Unless this can be overcome, the most profitable step forward is likely to be the development of a means of boosting the hepatic synthesis of PON1 whilst retaining the physiological mechanism by which it enters the hydrophobic lipid domains of HDL. An alternative is to increase cofactors of PON1. Unfortunately, raising apoA1 levels by overexpressing *APOA1* has proved disappointing unless several copies are expressed [31]. On the other hand, decreasing the expression of myeloperoxidase or of proteins that displace PON1 from HDL, such as SAA or apoA11, may prove therapeutic.

## 7. Conclusions

The antioxidant role of HDL is well-established. The concept of pro- and anti-inflammatory and pro- and anti-atherogenic HDL is of considerable interest and appears to be driven by the capacity of HDL to interfere with the oxidative modification of proteins, which is itself largely due to PON1 and to the hydrophobic, protective environment provided by HDL.
